# Product inhibition of cellulases studied with ^14^C-labeled cellulose substrates

**DOI:** 10.1186/1754-6834-6-104

**Published:** 2013-07-24

**Authors:** Hele Teugjas, Priit Väljamäe

**Affiliations:** 1 Institute of Molecular and Cell Biology, University of Tartu, Riia 23b – 202, Tartu 51010, Estonia

**Keywords:** Cellulase, Cellulose, Cellobiose, Glucose, Inhibition, *Acremonium thermophilum*, *Thermoascus aurantiacus*, *Chaetomium thermophilum*, *Trichoderma reesei*

## Abstract

**Background:**

As a green alternative for the production of transportation fuels, the enzymatic hydrolysis of lignocellulose and subsequent fermentation to ethanol are being intensively researched. To be economically feasible, the hydrolysis of lignocellulose must be conducted at a high concentration of solids, which results in high concentrations of hydrolysis end-products, cellobiose and glucose, making the relief of product inhibition of cellulases a major challenge in the process. However, little quantitative information on the product inhibition of individual cellulases acting on cellulose substrates is available because it is experimentally difficult to assess the hydrolysis of the heterogeneous polymeric substrate in the high background of added products.

**Results:**

The cellobiose and glucose inhibition of thermostable cellulases from *Acremonium thermophilum*, *Thermoascus aurantiacus*, and *Chaetomium thermophilum* acting on uniformly ^14^C-labeled bacterial cellulose and its derivatives, ^14^C-bacterial microcrystalline cellulose and ^14^C-amorphous cellulose, was studied. Cellulases from *Trichoderma reesei* were used for comparison. The enzymes most sensitive to cellobiose inhibition were glycoside hydrolase (GH) family 7 cellobiohydrolases (CBHs), followed by family 6 CBHs and endoglucanases (EGs). The strength of glucose inhibition followed the same order. The product inhibition of all enzymes was relieved at higher temperatures. The inhibition strength measured for GH7 CBHs with low molecular-weight model substrates did not correlate with that measured with ^14^C-cellulose substrates.

**Conclusions:**

GH7 CBHs are the primary targets for product inhibition of the synergistic hydrolysis of cellulose. The inhibition must be studied on cellulose substrates instead of on low molecular-weight model substrates when selecting enzymes for lignocellulose hydrolysis. The advantages of using higher temperatures are an increase in the catalytic efficiency of enzymes and the relief of product inhibition.

## Background

Cellulose is the most abundant biopolymer on Earth and has great potential as a renewable energy source. In nature, cellulose is degraded mainly by fungi and bacteria, which secrete cellulolytic enzymes [1]. These enzymes include cellulases, hemicellulases, and enzymes involved in lignin breakdown. Cellulases are divided into cellobiohydrolases (CBHs), endoglucanases (EGs) and β-glucosidases (BGs). CBHs are processive enzymes that liberate consecutive cellobiose units from cellulose chain ends, whereas EGs non-processively attack cellulose chains at random positions. β-Glucosidases hydrolyze cellobiose to glucose, thus relieving the product inhibition of CBHs [2]. One of the most efficient and best-characterized cellulolytic systems is that of the soft rot fungus *Tricoderma reesei* (*Tr*). The major component of the *Tr* cellulolytic system is the glycoside hydrolase (GH) family 7 [3,4] CBH, *Tr*Cel7A (formerly CBH I). *Tr* also secretes a less abundant CBH, *Tr*Cel6A (CBH II), and a number of EGs, including *Tr*Cel7B, *Tr*Cel5A and *Tr*Cel12A (EG I, EG II and EG III, respectively).

Cellulases are used in many biotechnological applications, such as fiber modification in the paper and textile industries, but they also have great potential in the emerging industry of ethanol production from lignocellulose. To decrease the water consumption and reduce the costs of equipment and distillation, the hydrolysis of lignocellulose must be conducted at a high concentration of solids. This approach inevitably results in high concentrations of the hydrolysis end-products cellobiose and glucose, and it has been proposed that the end-product inhibition of cellulases is rate limiting for lignocellulose hydrolysis in high-solid conditions [5]. Thus, relieving the product inhibition is a major challenge in the process, as well as in enzyme engineering [6]. The end-product inhibition can be relieved in a simultaneous saccharification and fermentation process, where the fermenting organism is added in parallel with hydrolytic enzymes, but one drawback is the need for different conditions for optimal hydrolysis and fermentation. The optimal temperature for yeast fermentation is approximately 35°C, whereas temperatures near 50°C are optimal for the performance of cellulases. A process concept using high temperature liquefaction with thermostable enzymes preceding simultaneous saccharification and fermentation has been developed [7], and this has triggered the search for novel thermostable enzymes [8,9].

Despite intensive efforts, little quantitative information about the end-product inhibition of cellulases is available. Many of the studies can be classified as “semi-quantitative”. Most often, the rates of cellulose hydrolysis measured in the presence and absence of β-glucosidase are compared [10-13]. In some studies, the experimental setup enabling the continuous elimination of end-products has been used [6]. The numerical values of inhibition constants have been obtained by the fitting of hydrolysis data to the complex equations derived for the full time-course [14-20]. The validity of these figures depends on the validity of the model [21]. Another problem lies in the possible interplay between parameters in trials, where values of multiple parameters are approximated by a single fit. The inhibition types reported include competitive, non-competitive, uncompetitive and mixed inhibition, whereas the values of inhibition constants vary over several orders of magnitude. One reason for the variation of reported inhibition types and the values of inhibition constants is that complex cellulase mixtures are often used instead of purified cellulase components in experiments. Different cellulase components may be inhibited to different extents and by different mechanisms, which clearly complicates the interpretation of the data. For literature reviews of earlier and more recent studies, see [22] and [23], respectively.

An inherent problem in measuring the strength of product inhibition is associated with difficulties in measuring the initial rates of product formation in the high background of the product added as an inhibitor. Three approaches can be used to overcome this: (i) measurement of the initial rates of substrate consumption instead of product formation [24]; (ii) measurement of the hydrolysis rate with a method that does not rely on measuring the concentration of the substrate or product; and (iii) the use of model substrates, whose conversion can be followed independently of the added products. Although emerging new methods, such as flow ellipsometry [25] and quartz crystal microbalance [26], enable the monitoring of changes in cellulose concentration in real time, these methods have not yet been applied to quantification of the inhibition of cellulases. The second approach has been applied for cellulases by following the rate of cellulose hydrolysis using isothermal titration calorimetry [27,28]. Because of the moderate standard enthalpy change of glycosidic bond hydrolysis, the low sensitivity is a drawback of calorimetry. While signal amplification systems can be used to measure cellulose hydrolysis, these systems are not applicable in studies of inhibition [29]. The third approach has been most widely used in studies of the inhibition of cellulases. The model substrates used can be divided into two classes, low-Mw and polymeric model substrates. Among low-Mw model substrates, the chromo- or fluorogenic derivatives of lactose or cellobiose are most often used [30]. However, these derivatives are not generally applicable. As an example, para-nitrophenyl-β-lactoside (pNPL) and 4-methylumbelliferyl-β-lactoside (MUL) are good substrates for GH7 CBHs such as *Tr*Cel7A and some EGs such as *Tr*Cel7B, but they are not hydrolyzed by GH6 CBHs such as *Tr*Cel6A. Another drawback of using low-Mw model substrates is that cellobiose inhibition appears to be much stronger with these substrates than with cellulose [31]. The reason for this may lie in different modes of action of cellulases on low-Mw model substrates and on cellulose [32] and in the experimental conditions used to measure enzyme inhibition [33]. Therefore, it is not possible to determine whether and to what extent the inhibition strength measured with low-Mw substrates reflects the inhibition strength with the real substrate, cellulose. Among polymeric model substrates, cellulose derivatives, in which hydroxyls are randomly substituted with chromo- or fluorophores (dyed cellulose), can be used [22,23]. The drawback of their use is that the tunnel-shaped active sites of CBHs cannot accommodate the bulky substitutes, and the application of these substrates is limited with EGs. Derivatives in which the reducing ends of cellulose are ^3^H-reduced to corresponding alditols have also been used [31]. The disadvantage of these substrates is that only the cleavage of reducing-end terminal glycosidic bonds can be measured. Therefore, these substrates are not applicable with non-reducing-end active CBHs such as *Tr*Cel6A. To overcome these limitations, we prepared uniformly ^14^C-labeled bacterial cellulose (^14^C-BC) by cultivating *Gluconobacterium xylinum* in the presence of ^14^C-glucose. ^14^C-BC and its derivatives, ^14^C-bacterial microcrystalline cellulose (^14^C-BMCC) and ^14^C-amorphous cellulose, were used to study the cellobiose and glucose inhibition of thermostable cellulases from *Acremonium thermophilum* (*At*), *Thermoascus aurantiacus* (*Ta*), and *Chaetomium thermophilum* (*Ct*)*.* Cellulases from these organisms have great potential in biotechnological applications [34-39]. Well-characterized cellulases from *Tr* were used for comparison.

## Results and discussion

### Measuring the strength of inhibition

The best parameter for describing the inhibitory strength of an inhibitor is *K*_i_, the equilibrium dissociation constant of an enzyme-inhibitor complex. *K*_i_ is a fundamental parameter of enzyme kinetics that is directly related to the thermodynamic stability of the enzyme-inhibitor complex. The conventional approach for the measurement of *K*_i_ involves the measurement of *k*_cat_ and *K*_M_ values for the substrate at different concentrations of an inhibitor. The plotting of *k*_cat_ and *K*_M_ or their combination as a function of inhibitor concentration allows the determination of both the type of inhibition and the *K*_i_ value. However, this approach is not applicable to cellulases acting on cellulose. The complex, multiple-mode binding of cellulases to the solid substrate obeys the so-called double-saturation character [1]. *K*_M_ values measured for cellulose depend on the enzyme concentration, and therefore, *K*_M_ has not its usual meaning. Because of the non-productive binding and strong time dependency, the measurement of the *k*_cat_ value is also not straightforward [40-42].

A simplified approach for assessing the inhibitory strength is to measure the *IC*_50_, the inhibitor concentration that halves the rate of the enzyme-catalyzed reaction. The *IC*_50_ is measured at one substrate concentration by varying the concentration of the inhibitor. Data are plotted as *v*_i_/*v*_0_ versus [*I*], where *v*_i_ and *v*_0_ are the rates measured in the presence and absence of inhibitor, respectively, and [*I*] is the concentration of inhibitor. To find the *IC*_50_, the data are first fitted to hyperbolae in the following form:

(1) $$\frac{v_{i}}{v_{0}}=\frac{\left[S\right]+C_{1}}{\left[S\right]+C_{1}\left(1+\frac{\left[I\right]}{C_{2}}\right)}$$

In the fitting of the data, the substrate concentration ([S]) is fixed to the value used in the experiments. The above value of [S] and the values of empirical constants *C*_1_ and *C*_2_ found by the fitting are further used to calculate the *IC*_50_ value using Equation 2:

(2) $$IC_{50}=C_{2}\left(1+\frac{\left[S\right]}{C_{1}}\right)$$

The *IC*_50_ is an empirical parameter and its value may depend on the concentration of the substrate (relative to its *K*_M_ value for the enzyme) used in the measurement of the *IC*_50_. If and how the *IC*_50_ value depends on [S]/*K*_M_ depends on the type of inhibition. In the case of competitive inhibition, the relationship among *IC*_50_, *K*_i_ and [S]/*K*_M_ is given as follows:

(3) $$IC_{50}=K_{i}\left(1+\frac{\left[S\right]}{K_{M}}\right)$$

Thus, if the inhibition is competitive and the [S] used in the measurement of the *IC*_50_ is well below its *K*_M_ value, the resulting *IC*_50_ value is close to the true *K*_i_ value. However, if [S] is near saturating for the enzyme, the inhibition appears to be weak, as the resulting *IC*_50_ is much higher than *K*_i_. The situation is opposite in the case of un-competitive inhibition, as in this case we have the following:

(4) $$IC_{50}=K_{i}\left(1+\frac{K_{M}}{\left[S\right]}\right)$$

In the case of mixed inhibition, the interplay among *IC*_50_, *K*_i_ (there are two different *K*_i_s now) and [S]/*K*_M_ is more complicated, and whether the inhibition appears to be stronger at low or high [S]/*K*_M_ ratio depends on which type of inhibition (competitive or un-competitive) is dominating. However, in the case of pure non-competitive inhibition, *IC*_50_ = *K*_i_, so *IC*_50_ represents the value of the true *K*_i_ at any substrate concentration used for its measurement.

### GH family 7 cellobiohydrolases

GH7 CBHs are major components of efficient fungal cellulase systems. They are processive enzymes that are responsible for the degradation of crystalline cellulose [43]. Because of their central role in cellulose degradation, the inhibition of GH7 CBHs is of utmost importance. Here, we undertook a study of the inhibition of GH7 CBHs acting on ^14^C-BC. Thermostable GH7 CBHs *At*Cel7, *Ta*Cel7A, and *Ct*Cel7A [44], along with *Tr*Cel7A, were characterized in terms of cellobiose and glucose inhibition. *T*_m_ values of 75°C, 69°C, 75°C and 65°C have been reported for *Ta*Cel7A, *At*Cel7A, *Ct*Cel7A and *Tr*Cel7A, respectively [44]. Although highly crystalline, the BC fiber contains a small fraction of heterogeneities [45,46]. These heterogeneities are preferentially degraded by cellulases, and their depletion is thought to be responsible for rate retardation of cellulose hydrolysis [47]. Thus, interpretation of the results of product inhibition is more straightforward if measured at a higher degree of substrate conversion. A very high degree of synergy between *Tr*Cel7A and EG has been reported with BC substrates [32,48,49]. To reach a higher degree of conversion and characterize the hydrolysis of bulk cellulose, the GH7 CBHs were thus provided with the EG, *Tr*Cel5A (10% on a molar basis).

**Figure 1:**
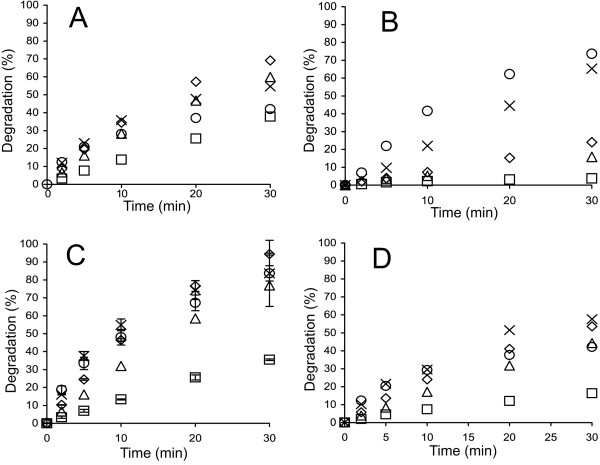
**Synergistic hydrolysis of **^**14**^**C-BC by GH7 CBHs at different temperatures. **^14^C-BC (0.25 mg ml^-1^) was incubated with 0.25 μM CBH, supplemented with 0.025 μM EG (*Tr*Cel5A) and 0.06 μM *N188*BG, at 25°C (□), 35°C (Δ), 40°C (◊), 50°C (×), and 60°C (○). CBH was **(A)***Tr*Cel7A, **(B)***Ta*Cel7A, **(C)***At*Cel7A, and **(D)***Ct*Cel7A.

**Figure 2:**
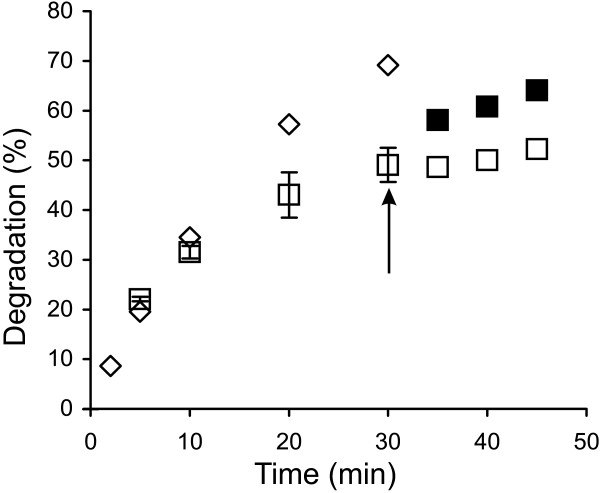
**Irreversible inactivation of *Tr*Cel7A is not responsible for the decreased hydrolysis rates at higher temperatures. **^14^C-BC (0.25 mg ml^-1^) was incubated with 0.25 μM *Tr*Cel7A, supplemented with 0.025 μM EG (*Tr*Cel5A) and 0.06 μM *N188*BG. Temperature was 40°C (◊) and 55°C (□). In one trial, the hydrolysis was conducted at 55°C for 30 min, and then the temperature was decreased (indicated by arrowhead) to 40°C (■).

Figure 1 shows the time courses for the synergistic hydrolysis of ^14^C-BC by CBHs (supplemented with *Tr*Cel5A and β-glucosidase, *N188*BG) at different temperatures. With all CBHs, the time courses of ^14^C-BC degradation measured at lower temperatures (25°C – 35°C) were nearly linear, whereas the time courses measured at higher temperatures gradually deviated from linearity. With *Ta*Cel7A as an exception, the degree of conversion after 30 min of hydrolysis measured at 60°C was less than that measured at 50°C (Figure 1). However, such a decrease in the degree of conversion with increasing temperature was not observed after 5 min of hydrolysis. Similar observations have also been made for the hydrolysis of pre-treated lignocellulose [36], suggesting that this phenomenon is not ^14^C-BC specific. The simplest explanation would be the thermal inactivation of enzymes that progresses with time. We tested the possible thermal inactivation of enzymes in an experiment where the hydrolysis began at 55°C, and after 30 min, the temperature was decreased to 40°C. *Tr*Cel7A was used as the CBH because of its lowest *T*_m_ value among the CBHs studied. Figure 2 demonstrates that despite a 15°C drop in temperature, the rate of cellulose hydrolysis actually increased. This finding rules out the irreversible inactivation of enzymes as the primary cause of the non-linearity in time curves observed at higher temperatures. However, the contribution of the reversible denaturation of enzymes cannot be ruled out. The hydrolysis of cellulose by CBH is a multi-step process including binding to cellulose, the capture of the cellulose chain-end, processive degradation, and dissociation [32,50]. Therefore, another possibility is that some kinetic property of CBHs is negatively affected by temperature. Whatever the underlying mechanisms, the change in the linearity of time curves depending on temperature may also result in a change in the apparent inhibition strength with hydrolysis time.

**Figure 3:**
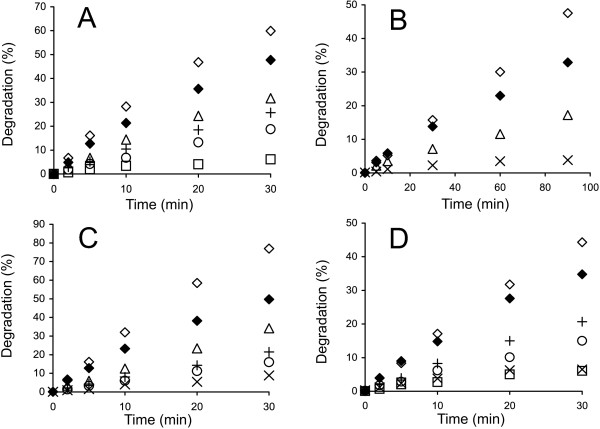
**Inhibition of GH7 CBHs by cellobiose. **^14^C-BC (0.25 mg ml^-1^) was incubated with a mixture of 0.25 μM CBH and 0.025 μM EG (*Tr*Cel5A) at 35°C. The concentration of cellobiose added was 0 mM + 0.06 μM *N188*BG (◊), 0 mM (♦), 0.5 mM (Δ), 1.0 mM (+), 2.0 mM (○), 5.0 mM (×) or 10 mM (□). CBH was **(A)***Tr*Cel7A, **(B)***Ta*Cel7A, **(C)***At*Cel7A, and **(D)***Ct*Cel7A.

**Figure 4:**
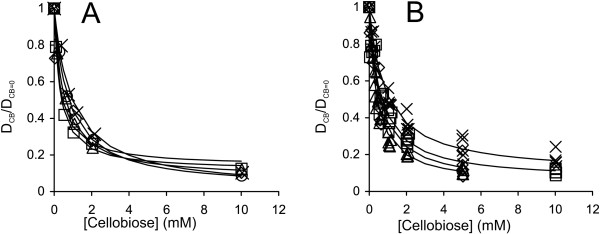
**Analysis of the inhibition of GH7 CBHs by cellobiose. (A)** Data for the hydrolysis of ^14^C-BC by the mixture of *Tr*Cel7A and *Tr*Cel5A at 35°C (Figure 3A) were rearranged in the coordinates (D_CB_/D_CB=0_) versus [cellobiose], where D_CB_ and D_CB=0_ represent the degree of conversion of ^14^C-BC in the presence and absence of cellobiose, respectively. The ratio of (D_CB_/D_CB=0_) was found after different times of hydrolysis, which were 2 min (◊), 5 min (□), 10 min (Δ), 20 min (○), and 30 min (×). **(B)** Data for the hydrolysis of ^14^C-BC by the mixture of CBH and *Tr*Cel5A at 35°C (Figure 3) in the coordinates (D_CB_/D_CB=0_) versus [cellobiose]. (D_CB_/D_CB=0_) values for all hydrolysis time points are shown. CBH was *Tr*Cel7A (□), *Ta*Cel7A, (◊), *At*Cel7A (Δ), and *Ct*Cel7A (×). Solid lines are from the non-linear regression according to Equation 5.

To study the cellobiose inhibition of GH7 CBHs, the synergistic hydrolysis of ^14^C-BC in the presence of added cellobiose was followed (Figure 3, Additional file 1: Figures S1 and S2). Because the cellobiose inhibition of the EG *Tr*Cel5A is much weaker than that of GH7 CBHs [28,31], the inhibition of synergistic hydrolysis apparently reflects the inhibition of CBH. The strength of cellobiose inhibition was analyzed using plots of (D_CB_/D_CB=0_) versus [cellobiose], where D_CB_ and D_CB=0_ represent the degree of conversion of ^14^C-BC in the presence and absence of cellobiose, respectively (Figure 4, Additional file 1: Figure S3). In the case of experiments without added cellobiose, the reactions were provided with *N188*BG to prevent the inhibition of the CBH by the cellobiose released during hydrolysis. Experiments with no added cellobiose and without BG were also conducted. Comparison of the results obtained with and without *N188*BG (both without added cellobiose) demonstrates that the inhibition of CBHs by the cellobiose released during hydrolysis was significant (Figure 3, Additional file 1: Figures S1 and S2). Therefore, the concentration of the cellobiose released during hydrolysis was added to the concentration of externally supplied cellobiose in generating the plots in Figure 4 and Additional file 1: Figure S3. For the calculation of *IC*_50_ values, the data were first fitted to hyperbolae:

(5) DCBDCB=0=C14BC+C11−HC14BC+C11+CBC2+H

where [CB] is the concentration of cellobiose; [^14^CBC] is the concentration of ^14^C-BC used in the experiment; and *C*_1_, *C*_2_ and *H* are empirical constants. Equation 5 differs from Equation 1 by the presence of constant *H*. *H* was included to improve the fit and is a constant that accounts for the background radioactivity (the degree of conversion that is independent of CBH). The degree of conversion resulting from the activity of the EG was measured in a separate experiment (Additional file 1: Figure S4) and was subtracted from the degree of conversion resulting from the synergistic hydrolysis. Thus, in the case of complete inhibition, *H* should have a value of zero. Non-zero *H* values are indicative of partial inhibition. However, because the *H* values remained between 0 and 0.2 and were even negative in some cases, they may also be a result of experimental uncertainty. Provided with the values of *C*_1_, *C*_2_ and *H*, the value of *IC*_50_ was calculated as follows:

(6) IC50=C14BC+C1C1C21−2H

Using the time course data measured in the presence and absence of cellobiose, the *IC*_50_ values were first found separately for each time point (Figure 4A) [32]. Table 1 lists the average *IC*_50_ values over all time points. In some cases, a systematic drift of *IC*_50_ values with hydrolysis time was observed, which may indicate that different rate-limiting factors with different *IC*_50_ values may control the hydrolysis rate in different time or conversion frames. An apparent decrease in inhibition strength with increasing hydrolysis time was also observed for the cellobiose inhibition of EG *Tr*Cel7B [28]. A more systematic analysis of the time dependency of *IC*_50_ values remained outside the scope of the present study. The enzyme most sensitive to cellobiose inhibition appeared to be *Ta*Cel7A, followed by *At*Cel7A, *Tr*Cel7A and *Ct*Cel7A (Table 1). However, the differences between enzymes were not very prominent, especially considering error limits. With all enzymes, the strength of cellobiose inhibition decreased significantly with increasing temperature.

**Table 1:** **Inhibition of GH7 CBHs by cellobiose and glucose studied with **^**14**^**C-BC substrate**

	***IC***_**50 **_**for cellobiose (mM)**			***IC***_**50 **_**for glucose (mM)**
	25°C	35°C	50°C	35°C
*Tr*Cel7A	0.38 ± 0.03^a^	0.68 ± 0.24^b^	2.61 ± 0.10	420 ± 230^c^
*At*Cel7A	0.19 ± 0.10^c^	0.44 ± 0.10	2.12 ± 1.40^b^	420 ± 180^c^
*Ct*Cel7A	0.41 ± 0.06	1.08 ± 0.22	2.48 ± 0.91^b^	360 ± 170^c^
*Ta*Cel7A		0.58 ± 0.35	0.93 ± 0.10	

^a^ Data from [32].

^b^*IC*_50_ increased with hydrolysis time.

^c^*IC*_50_ decreased with hydrolysis time.

**Figure 5:**
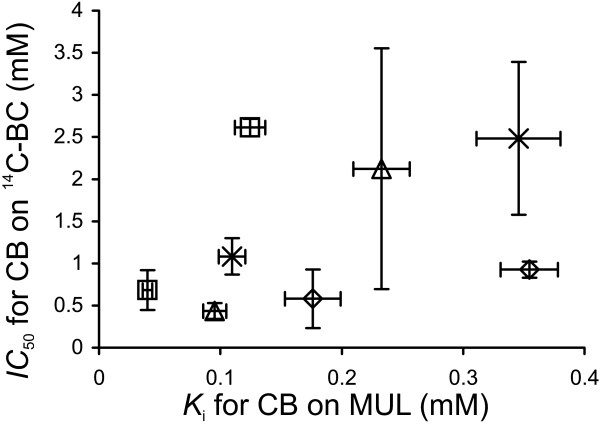
**Relative strength of cellobiose inhibition of GH7 CBHs depends on the substrate.***K*_i_ values measured for MUL hydrolysis and *IC*_50_ values measured for the hydrolysis of ^14^C-BC, both at 35°C and 50°C, were taken from Table 2 and Table 1, respectively. CBH was *Tr*Cel7A (□), *Ta*Cel7A, (◊), *At*Cel7A (Δ), and *Ct*Cel7A (×).

The cellobiose inhibition of GH7 CBHs is most often studied on low-Mw model substrates. However, it has been shown that the inhibition of CBHs acting on low-Mw substrates appears to be much stronger than that on cellulose substrates [31,33]. The *K*_i_ values for cellobiose inhibition of GH7 CBHs measured on low-Mw substrates are in the micromolar range [44,51,52], whereas those measured on cellulose are in the low- to high-millimolar range [28,31,32]. An interesting exception is Cel7A from *Trichoderma harzianum*, which shows a 7.2 mM *K*_i_ value for the cellobiose inhibition of the hydrolysis of chloro-nitrophenyl lactoside [53]. Unfortunately, the inhibition of this enzyme on cellulose has not been studied. We also studied the cellobiose inhibition of GH7 CBHs acting on MUL. The initial rates of MUL hydrolysis measured in the presence and absence of added cellobiose were first analyzed according to Equation 1, and the *IC*_50_ values were found using Equation 2. As cellobiose was shown to be a competitive inhibitor for these CBHs acting on MUL [44] and the concentration of MUL used in the experiments (5 μM) was far below its *K*_M_ value (approximately 300 μM [44]), the measured *IC*_50_ value represents the true *K*_i_ (see Equation 3). The resulting *K*_i_ values are listed in Table 2. Van´t Hoff analysis of the temperature dependency of the *IC*_50_ and *K*_i_ values of *Tr*Cel7A resulted in standard enthalpy changes of 63.6 ± 2.6 kJ mol^-1^ (for *IC*_50_ values on ^14^C-BC, Table 1) and approximately 63 kJ mol^-1^ (for *K*_i_ values on MUL, Table 2). The inhibition of MUL hydrolysis is attributable to the binding of cellobiose to the product sites (+1/+2) of *Tr*Cel7A [52]. Similar standard enthalpy changes thus suggest that the cellobiose inhibition of the synergistic hydrolysis of ^14^C-BC is also attributable to the binding of cellobiose to sites +1/+2. Nonetheless, for all CBHs, the *K*_i_ values found for the cellobiose inhibition of MUL hydrolysis (Table 2) were smaller than the corresponding *IC*_50_ values for the inhibition of ^14^C-BC hydrolysis (Table 1). The reason for this difference may lie in the different modes of action used by CBHs with low-M_w_ model substrates and cellulose and therefore the different types of inhibition [32]. Another possible explanation is that the cellobiose inhibition of CBHs on cellulose is competitive and that the concentration of cellulose chain ends used in the measurement of the *IC*_50_ value is higher than the corresponding *K*_M_ value. In this case, the observed *IC*_50_ is expected to be higher than the *K*_i_ (see Equation 3), and the inhibition of cellulose hydrolysis appears to be weak. This scenario has been proposed to explain the differences in the inhibitory strength of xylo-oligosaccharides toward CBHs acting on MUL and cellulose [33]. The binding of xylo-oligosaccharides with DP 8 – 10 is expected to mimic the binding of the cellulose chain to the active site of *Tr*Cel7A, resulting in competitive inhibition. In contrast, despite the strong binding of cellobiose to the product sites (+1/+2) of *Tr*Cel7A [52,54,55], the cellulose chain can still bind to the substrate sites (from −7 to −1), and this predicts non-competitive inhibition [23,31,32]. The results of our previous studies of the inhibition of *Tr*Cel7A under single-turnover and steady-state conditions suggested that cellobiose might be a mixed-type inhibitor of *Tr*Cel7A acting on cellulose. The binding of cellobiose to the product and substrate binding sites was proposed to be responsible for the non-competitive and competitive components of inhibition, respectively [32]. Observations that the binding affinity of *Tr*Cel7A and *Tr*Cel6A towards cellulose increased in the presence of cellobiose also suggest an inhibition mode that is not competitive [56,57]. From the practical point of view, it is important to note that for different CBHs, the differences in inhibition strength observed on MUL and cellulose were not of the same magnitude (Figure 5). This result can be exemplified best by *Ta*Cel7A, which appeared to be most resistant to cellobiose inhibition on MUL substrate (Table 2) but was most sensitive to cellobiose inhibition on cellulose (Table 1). This finding stresses the importance of the use of “as native as possible” screening systems for selecting cellulases [58].

**Table 2:** Inhibition of GH7 CBHs by cellobiose studied with MUL substrate

	***K***_**i **_**for cellobiose (mM)**	
	35°C	50°C
*Tr*Cel7A	0.040	0.124
*At*Cel7A	0.095	0.233
*Ct*Cel7A	0.110	0.346
*Ta*Cel7A	0.176	0.355

**Figure 6:**
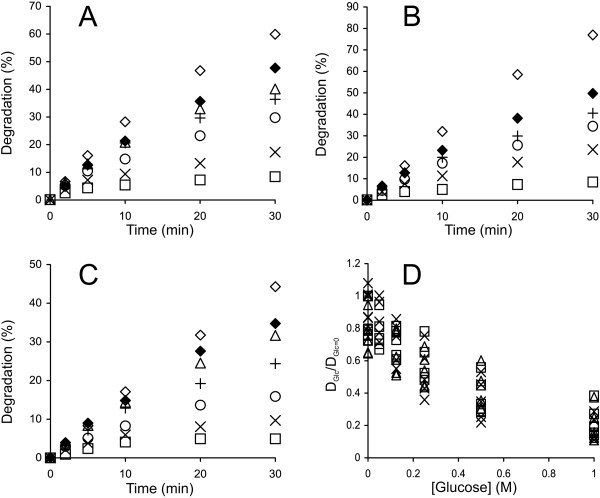
**Inhibition of GH7 CBHs by glucose. **^14^C-BC (0.25 mg ml^-1^) was incubated with a mixture of 0.25 μM CBH and 0.025 μM EG (*Tr*Cel5A) at 35°C. The concentration of added glucose was as follows: 0 M + 0.06 μM *N188*BG (◊), 0 M (♦), 0.05 M (Δ), 0.125 M (+), 0.25 M (○), 0.5 M (×) or 1.0 M (□). CBH was as follows: **(A)***Tr*Cel7A, **(B)***At*Cel7A, and **(C)***Ct*Cel7A. **(D)** Hydrolysis data in the coordinates (D_Glc_/D_Glc=0_) versus [glucose], where D_Glc_ and D_Glc=0_ represent the degree of conversion of ^14^C-BC in the presence and absence (+*N188*BG series) of added glucose, respectively. (D_Glc_/D_Glc=0_) values for all hydrolysis time points are shown. CBH was *Tr*Cel7A (□), *At*Cel7A (Δ), and *Ct*Cel7A (×).

The glucose inhibition of CBHs with ^14^C-BC as the substrate was also studied. CBHs were provided with EG *Tr*Cel5A (*Tr*Cel5A is not significantly inhibited by glucose [28]) and also with BG in the experiments without added glucose. The time courses of ^14^C-BC hydrolysis in the presence and absence of added glucose are shown in Figure 6. As revealed by the scattering of data points in the plot of (D_Glc_/D_Glc=0_) versus [glucose] (Figure 6D), the inhibition by the cellobiose released during hydrolysis was significant. This result was accounted for by adding the term [CB]/*IC*_50(CB)_ ([CB] is the concentration of the released cellobiose, and *IC*_50(CB)_ is the *IC*_50_ for cellobiose previously determined) to Equation 5 to create Equation 7:

(7) DGlcDGlc=0=C14BC+C11−HC14BC+C11+CBIC50CB+GlcC2+H

D_Glc_ and D_Glc=0_ represent the degree of conversion of ^14^C-BC in the presence and absence of added glucose, respectively; [Glc] is the concentration of added glucose; [^14^CBC] is the ^14^C-BC concentration used in the experiment; and *C*_1_, *C*_2_ and *H* are empirical constants. The values of *C*_1_, *C*_2_ and *H* obtained by the fitting of the data to Equation 7 were used to calculate the *IC*_50_ for glucose according to Equation 6. The glucose inhibition of GH7 CBHs was more than two orders of magnitude weaker than cellobiose inhibition (Table 1). Although relatively weak, glucose inhibition may become significant in the separate hydrolysis and fermentation of lignocellulose at a high dry matter consistency, where glucose may accumulate to well above 50 g/l (0.28 M) [5,23].

### GH family 6 cellobiohydrolases

GH6 CBHs are the second most abundant components of fungal cellulase systems. They are inverting CBHs that preferentially attack cellulose chains from non-reducing ends. To date, there are no good chromo- or fluorogenic model substrates for GH6 CBHs [59]. Because of the different chain-end preferences, inhibition studies on reducing-end-labeled cellulose substrates are also not applicable [31]. Therefore, little is known about the strength of the product inhibition of GH6 CBHs. From the reported binding constants measured using fluorophore competition experiments [60,61] and analysis of the progress curves of cellotriose hydrolysis [51,62], *K*_i_ values in a sub- to low-millimolar range can be calculated for the interaction of *Tr*Cel6A with cellobiose and glucose.

**Figure 7:**
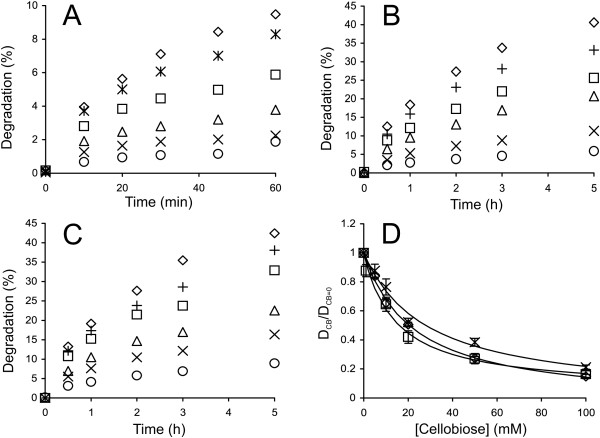
**Inhibition of GH6 CBHs by cellobiose. (A) **^14^C-BC (0.25 mg ml^-1^) was incubated with a mixture of 0.25 μM *Tr*Cel6A and 0.025 μM EG (*Tr*Cel5A) at 25°C. **(B** and **C) **^14^C-BMCC (0.25 mg ml^-1^) was incubated with 0.25 μM *Tr*Cel6A (panel B) or with 0.25 μM *Ct*Cel6A **(**panel **C)** at 50°C. The concentration of added cellobiose was 0 mM + 0.06 μM *N188*BG (◊), 1.0 mM (*), 5.0 mM (+), 10 mM (□), 20 mM (Δ), 50 mM (×), or 100 mM (○). **(D)** Hydrolysis data (from panels A-C) in the coordinates (D_CB_/D_CB=0_) versus [cellobiose], where D_CB_ and D_CB=0_ represent the degree of conversion of ^14^C-cellulose in the presence and absence of cellobiose, respectively. The average (D_CB_/D_CB=0_) values over hydrolysis time points are plotted. Solid lines are from the non-linear regression according to Equation 5. *Tr*Cel6A + *Tr*Cel5A on ^14^C-BC (□), *Tr*Cel6A on ^14^C-BMCC (◊), and *Ct*Cel6A on ^14^C-BMCC (×).

**Figure 8:**
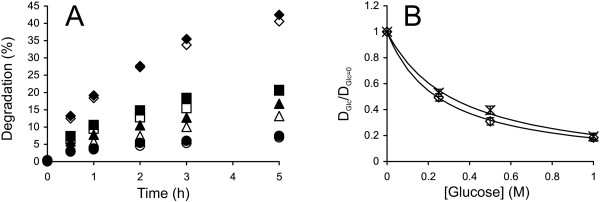
**Inhibition of GH6 CBHs by glucose. (A) **^14^C-BMCC (0.25 mg ml^-1^) was incubated with 0.25 μM *Tr*Cel6A (opened symbols) or with 0.25 μM *Ct*Cel6A (filled symbols) 50°C. The concentration of added glucose was as follows: 0 M + 0.06 μM *N188*BG (◊,♦), 0.25 M (□, ■), 0.5 M (Δ, ▲), or 1.0 M (○, ●). **(B)** Hydrolysis data in coordinates (D_Glc_/D_Glc=0_) *versus* [glucose] where D_Glc_ and D_Glc=0_ represent the degree of conversion of ^14^C-BMCC in the presence and absence of added glucose, respectively. (D_Glc_/D_Glc=0_) values for all hydrolysis time points are shown. Solid lines are from the non-linear regression according to Equation 5 (the terms referring to cellobiose were replaced with corresponding terms for glucose). CBH was *Tr*Cel6A (◊) or *Ct*Cel6A (×).

Here, we characterized the cellobiose and glucose inhibition of *Tr*Cel6A and its thermophilic counterpart, *Ct*Cel6A [9,63]. First, the cellobiose inhibition of the synergistic hydrolysis of ^14^C-BC by *Tr*Cel6A and *Tr*Cel5A was studied (Figure 7A). As *Tr*Cel6A was less sensitive to cellobiose inhibition than *Tr*Cel7A, the contribution of the cellobiose released during hydrolysis was not significant, and an average (D_CB_/D_CB=0_) over all time points was used in plotting (D_CB_/D_CB=0_) versus [cellobiose] (Figure 7D). No significant systematic variation of D_CB_/D_CB=0_ depending on hydrolysis time was observed. As in the case of GH7 CBHs, the *IC*_50_ value was found using Equations 5 and 6. Because the *IC*_50_ value for synergistic hydrolysis (Table 3) was of the same order as the apparent *K*_i_ value reported for *Tr*Cel5A [31], we further tested the inhibition of individual *Tr*Cel6A. BC is not a good substrate for *Tr*Cel6A, but its acid-treated derivative, BMCC, is readily degraded by the enzyme. Therefore, we prepared ^14^C-BMCC by the heterogeneous acid hydrolysis of ^14^C-BC. The time courses of ^14^C-BMCC hydrolysis by *Tr*Cel6A and *Ct*Cel6A are shown in Figures 7B and 7C. Without supplied cellobiose, both enzymes had similar activity with the ^14^C-BMCC substrate, but *Ct*Cel6A was somewhat more resistant to cellobiose inhibition (Figure 7D, Table 3). The *IC*_50_ value for *Tr*Cel6A by itself was similar to that found for the synergistic hydrolysis. This result suggests that the inhibition of *Tr*Cel6A was responsible for the cellobiose inhibition of the synergistic hydrolysis of ^14^C-BC. The glucose inhibition of *Tr*Cel6A and *Ct*Cel6A with ^14^C-BMCC as a substrate was also studied (Figure 8). Because the inhibition by cellobiose released during hydrolysis was not significant, a simpler equation, Equation 5 (the terms referring to cellobiose were replaced with corresponding terms for glucose), was used instead of Equation 7 to analyze the glucose inhibition of GH6 CBHs. Glucose appeared to be an approximately 10 times weaker inhibitor of *Tr*Cel6A and *Ct*Cel6A than cellobiose (Table 3), but comparison with corresponding figures for GH7 CBHs (Table 1) reveals that glucose is a relatively stronger inhibitor of GH6 than GH7 CBHs. The same result was also observed in a recent calorimetry study of the inhibition of *Tr* cellulases acting on amorphous cellulose [28]. However, the *IC*_50_ values found by Murphy et al. [28] for the cellobiose inhibition of *Tr*Cel7A and *Tr*Cel6A were approximately one order of magnitude higher than ours. Whether the differences in the strengths of cellobiose inhibition reflect the differences in substrates or the methods used for the measurement of inhibition is not known. Comparison of the *IC*_50_ values measured here with *K*_i_ values derived from binding constants measured using low-Mw substrates and ligands as competitors [60-62] reveals the same trend as in the case of GH7 CBHs: the binding of cellobiose and glucose appears to be weaker when assessed on polymeric substrates.

**Table 3:** **Inhibition of GH6 CBHs by cellobiose and glucose studied with **^**14**^**C-BC and **^**14**^**C-BMCC substrates**

	***IC***_**50 **_**(mM)**	
	Cellobiose	Glucose
*Tr*Cel6A ^a^	16 ± 0.5	
*Tr*Cel6A ^b^	20 ± 1.4	240 ± 26
*Ct*Cel6A ^b^	28 ± 4.5	301 ± 30

^a^ Synergistic hydrolysis of ^14^C-BC by *Tr*Cel6A and EG, *Tr*Cel5A, at 25°C.

^b^ Hydrolysis of ^14^C-BMCC by GH6 CBH at 50°C.

### Endoglucanases

**Figure 9:**
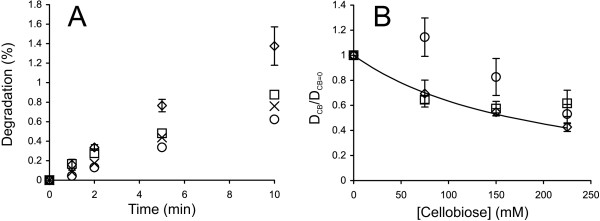
**Inhibition of EGs by cellobiose. (A) **^14^C-amorphous cellulose (0.5 mg ml^-1^) was incubated with 2.5 nM *Tr*Cel7B at 35°C. The concentration of added cellobiose was 0 mM (◊), 75 mM (□), 150 mM (×), or 225 mM (○). **(B)** Hydrolysis data from panel A and Additional file 1: Figure S5 in the coordinates (D_CB_/D_CB=0_) versus [cellobiose] where D_CB_ and D_CB=0_ represent the degree of conversion of ^14^C-cellulose in the presence and absence of cellobiose, respectively. Average (D_CB_/D_CB=0_) values over hydrolysis time points are plotted. The solid line is from the non-linear regression according to Equation 5. *Tr*Cel7B (◊), *Tr*Cel5A (□), and *Tr*Cel12A (○).

EGs are a diverse group of enzymes present in all efficient cellulase systems. Their best recognized function is their synergism with CBHs. Depending on the conditions, the degree of synergistic effect may be more than 10-fold [32,64]. Therefore, the inhibition of the EG component may result in a drastic decrease in the rate of the synergistic hydrolysis of cellulose. The main soluble product of the EG-catalyzed cellulose hydrolysis is cellobiose, but some glucose and higher-order oligosaccharides are also produced [65]. Here, we studied the cellobiose inhibition of the EGs *Tr*Cel7B, *Tr*Cel5A and *Tr*Cel12A with ^14^C-amorphous cellulose substrate. The enzyme concentrations and hydrolysis times were adjusted so that the linear region of the time course was studied. The time courses for the hydrolysis of ^14^C-amorphous cellulose by *Tr*Cel7B in the presence and absence of added cellobiose are shown in Figure 9A. For the results with *Tr*Cel5A and *Tr*Cel12A, see Additional file 1: Figure S5. The “conventional” inhibition pattern was observed only in the case of *Tr*Cel7B, with an *IC*_50_ value of 168 ± 2 mM. This figure is reasonably well in line with that measured for *Tr*Cel7B on amorphous cellulose using isothermal titration calorimetry [28]. Calorimetry measures the amount of glycosidic bonds that are cleaved irrespective of the solubility of the products [41]. Thus, the agreement between the *IC*_50_ values from calorimetric measurements and those reported here suggests that the inhibition of the release of soluble products represents the inhibition of the total activity of *Tr*Cel7B. However, we have previously reported an apparent *K*_i_ value of 11 ± 3 mM for *Tr*Cel7B with a ^3^H-reduced amorphous cellulose substrate [31]. Thus, the cellobiose inhibition of *Tr*Cel7B on uniformly ^14^C-labeled amorphous cellulose was much weaker. The same was also true for *Tr*Cel5A. The inhibition of *Tr*Cel5A and *Tr*Cel12A was not accountable by Equation 5 (Figure 9B). In the case of *Tr*Cel5A, the initial drop in activity was followed by a slight increase at the highest cellobiose concentration tested. In the case of *Tr*Cel12A, there was an apparent activation at a lower cellobiose concentration of 75 mM, followed by a decrease in activity with increasing cellobiose concentration (Figure 9B). We previously observed the apparent activation of *Tr*Cel12A in the cellobiose concentration range of 1 mM – 100 mM acting on a ^3^H-reduced amorphous cellulose substrate [31]. Glucose concentration dependent apparent activation or inhibition of pNPG-ase activity of BGs has also been observed [66-70]. The concentration-dependent apparent activation or inhibition most likely reflects the complex kinetics with competing hydrolytic and transglycosylation reactions [28,31]. Whether the sugar appears to be an inhibitor or an activator may depend on the rate-limiting step, which may also change depending on the sugar concentration and the experiment conditions, e.g., the method used for rate measurement. Although the *IC*_50_ values cannot be calculated for *Tr*Cel5A and *Tr*Cel12A, approximate figures in a few hundred millimolar range can be estimated by visual inspection of the data in Figure 9B. The *K*_i_ value of 424 μM has been reported for the cellobiose inhibition of *Tr*Cel5A acting on cellohexaose [27]. Thus, the strong dependence of inhibition strength on the type of substrate used seems to also be true for EGs. Despite some discrepancies in *IC*_50_ values, the inhibition of EGs is far weaker than that of CBHs and is not responsible for the cellobiose inhibition of synergistic hydrolysis.

## Conclusions

Our data presented here, together with those from the literature, strongly suggest that the inhibition of cellulases must be studied on cellulose substrates instead of on low-Mw model substrates. The enzymes most sensitive to cellobiose inhibition were GH7 CBHs, followed by GH6 CBHs and EGs. The strength of glucose inhibition followed the same order. Thus, the GH7 CBHs are primary targets for product inhibition of the synergistic hydrolysis of cellulose. With all enzymes, the strength of the product inhibition decreased with increasing temperature.

## Methods

### Materials

Glucose, MUL, pNPL, Novozyme®188, and BSA were purchased from Sigma-Aldrich. Cellobiose (≥ 99%) was from Fluka. D-[U-^14^C] glucose with a specific activity of 262 mCi mmol^-1^ was from Hartmann Analytic GmbH. Scintillation cocktail was from Merck. All chemicals were used as purchased.

### ^14^C-cellulose substrates

^14^C-BC was prepared by laboratory fermentation of the *Gluconobacter xylinum* strain ATCC 53582 [71] in the presence of [U-^14^C] glucose carbon source [32]. ^14^C-BC had a specific activity 450,000 DPM mg^-1^. ^14^C-BMCC was prepared by the limited acid hydrolysis of ^14^C-BC, and ^14^C-amorphous cellulose was prepared from ^14^C-BMCC by dissolution and regeneration from phosphoric acid [71]. The total concentration of cellulose was determined by the anthrone sulfuric acid method.

### Enzymes

*Tr*Cel7A was purified from the culture filtrate of *Tr* QM 9414 as described previously [72]. Culture filtrates containing *At*Cel7A, *Ct*Cel7A or *Ta*Cel7A were kindly provided by Terhi Puranen from Roal Oy (Rajamäki, Finland). CBHs were heterologously expressed in a *Tr* strain lacking the genes of four major cellulases [34,44]. The natively carbohydrate-binding module-less *Ta*Cel7A was provided with the carbohydrate binding module of *Tr*Cel7A [34,44]. CBHs were purified on a Q-Sepharose column after buffer exchange on a Toyopearl HW-40 column. For ion-exchange chromatography on Q-Sepharose, the column was equilibrated with 20 mM sodium phosphate, pH 6.0 (in the case of *At*Cel7A and *Ta*Cel7A) or with 20 mM sodium phosphate, pH 6.5 (in the case of *Ct*Cel7A). CBHs were eluted with a linear gradient of 0 – 0.3 M NaCl in equilibration buffer.

*Tr*Cel6A was purified from the culture filtrate of *Tr* QM 9414 as described previously [72,73]. The culture filtrate of *Ct*Cel6A heterologously expressed in *Tr* originated from Roal OY (Rajamäki, Finland) and was kindly provided by Matti Siika-Aho from VTT (Espoo, Finland). *Ct*Cel6A was purified on a DEAE-Sepharose column after buffer exchange on a Toyopearl HW-40 column. For ion-exchange chromatography on DEAE-Sepharose, the column was equilibrated with 20 mM sodium phosphate (pH 7.0), and *Ct*Cel6A was eluted with a linear gradient of 0 – 0.5 M NaCl in equilibration buffer.

*Tr*Cel7B, *Tr*Cel5A, and *Tr*Cel12A were purified from the culture filtrate of *Tr* QM 9414 as described previously [72,74,75]. *N188*BG was purified from Novozyme®188 as described in [76].

The concentration of the enzymes was measured from the absorbance at 280 nm using theoretical ϵ_280_ values.

### Activity and inhibition of GH7 CBHs

The activity and inhibition of GH7 CBHs were assessed by following the synergistic hydrolysis of ^14^C-BC. For that, ^14^C-BC (0.25 g l^-1^) was incubated (without stirring) with a mixture of CBH (0.25 μM), *Tr*Cel5A (0.025 μM) and *N188*BG (0.06 μM) in 50 mM sodium acetate buffer pH 5.0 containing BSA (0.1 g l^-1^). At selected times, 0.2-ml aliquots were withdrawn and added to 20 μl 1 M NaOH to stop the reaction. Residual cellulose was separated by centrifugation (2 min, 10^4^ x *g*), and radioactivity in the supernatant was quantified using liquid scintillation counting. The degree of cellulose degradation was calculated from the ratio of radioactivity in the supernatant to the total radioactivity in the hydrolysis mixture. In the case of inhibition studies, the reactions were supplied with cellobiose and glucose at different concentrations, and *N188*BG was omitted.

For the inhibition of enzyme acting on the low-Mw substrate, the initial rates of the hydrolysis of MUL in the presence and absence of added cellobiose were followed. MUL (5 μM) was incubated with CBH (10 nM) in 50 mM sodium acetate buffer, pH 5.0, containing BSA (0.1 g l^-1^). Reactions were stopped by the addition of NH_3_ (final concentration 0.1 M), and the released 4-methylumbelliferone was quantified by fluorescence using excitation and emission wavelengths of 360 nm and 450 nm, respectively.

### Activity and inhibition of GH6 CBHs

GH6 CBHs were assessed by observing the hydrolysis of ^14^C-BMCC. ^14^C-BMCC (0.25 g l^-1^) was incubated (with shaking at 350 rpm) with CBH (0.25 μM) and *N188*BG (0.06 μM) in 50 mM sodium acetate buffer, pH 5.0, containing BSA (0.1 g l^-1^). The remainder of the procedure was identical to that described for GH7 CBHs. In the case of inhibition studies, the reactions were supplied with cellobiose and glucose at different concentrations, and *N188*BG was omitted.

The cellobiose inhibition of the synergistic hydrolysis of ^14^C-BC was performed identically to the procedure described for GH7 CBHs, but the CBH component was 0.25 μM *Tr*Cel6A.

### Activity and inhibition of EGs

EGs were assessed on ^14^C-amorphous cellulose. ^14^C-amorphous cellulose (0.5 g l^-1^) was incubated (with shaking at 700 rpm) with EG in 50 mM sodium acetate buffer, pH 5.0, containing BSA (0.1 g l^-1^) in the presence and absence of added cellobiose. The concentration of EG was 2.5 nM, 5.0 nM, and 50 nM for *Tr*Cel7B, *Tr*Cel5A, and *Tr*Cel12A, respectively. The remainder of the procedure was identical to that described for GH7 CBHs.

## Abbreviations

At: *Acremonium thermophilum*; BG: β-glucosidase; BSA: Bovine serum albumin; CB: Cellobiose; 14C-BC: ^14^C-labeled bacterial cellulose; CBH: Cellobiohydrolase; 14C-BMCC: ^14^C-labeled bacterial microcrystalline cellulose; Ct: *Chaetomium thermophilum*; Di: Degree of conversion in the presence of inhibitor *i*; D0: Degree of conversion in the absence of inhibitor; DP: Degree of polymerization; EG: Endoglucanase; GH: Glycoside hydrolase; Glc: Glucose; MUL: 4-methylumbelliferyl-β-lactoside; pNP: Para-nitrophenol; pNPL: Para-nitrophenyl-β-lactoside; Ta: *Thermoascus aurantiacus*; Tr: *Trichoderma reesei*

## Competing interests

The authors declare that they have no competing interests.

## Authors’ contributions

HT and PV designed and performed the experiments. PV wrote the paper. Both authors read and approved the final manuscript.

## Supplementary Material

Additional file 1: Figure S1 Inhibition of GH7 CBHs by cellobiose at 50°C. Figure S2. Inhibition of GH7 CBHs by cellobiose at 25°C. Figure S3. Analysis of the inhibition of GH7 CBHs by cellobiose at 25°C and 50°C. Figure S4. Hydrolysis of ^14^C-BC by EG, *Tr*Cel5A. Figure S5. Inhibition of EGs, *Tr*Cel5A and *Tr*Cel12A, by cellobiose. Click here for file
