# Supplementary material for: Product inhibition of cellulases studied with 14C-labeled cellulose substrates
Source: Biotechnol Biofuels. 2013 Jul 24;6:104. doi: 10.1186/1754-6834-6-104 (PMC3726336; doi:10.1186/1754-6834-6-104)
Supplement: Additional file 1: Figure S1 — Inhibition of GH7 CBHs by cellobiose at 50°C. Figure S2. Inhibition of GH7 CBHs by cellobiose at 25°C. Figure S3. Analysis of the inhibition of GH7 CBHs by cellobiose at 25°C and 50°C. Figure S4. Hydrolysis of 14C-BC by EG, TrCel5A. Figure S5. Inhibition of EGs, TrCel5A and TrCel12A, by cellobiose. [file 1754-6834-6-104-S1.doc]

**Supplemental material**

**Product inhibition of cellulases studied with ^14^C-labeled cellulose substrates**

Hele Teugjas and Priit Väljamäe*

Institute of Molecular and Cell Biology, University of Tartu, Riia 23b – 202, 51010 Tartu, Estonia.

*Corresponding author: priit.valjamae@ut.ee


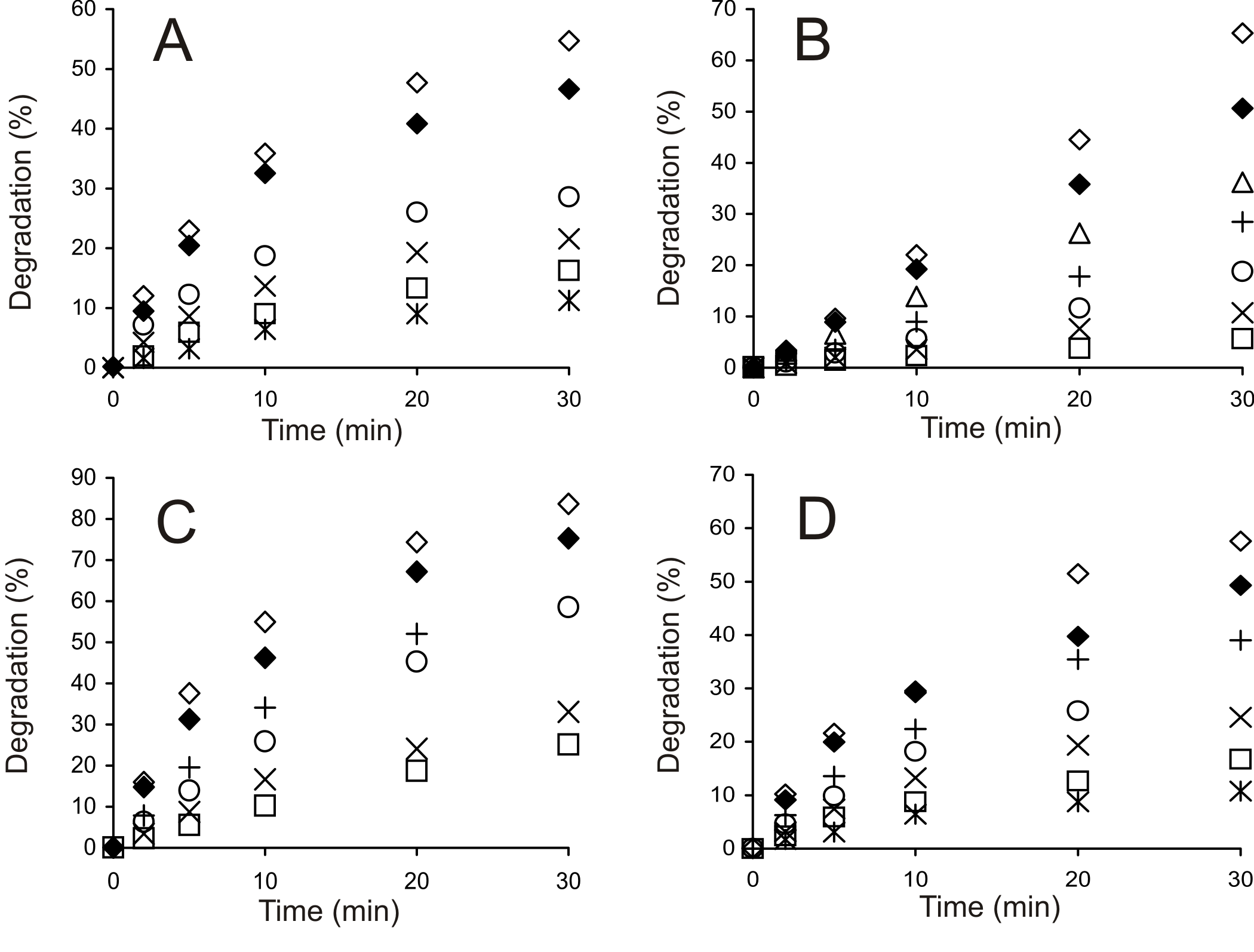


**Figure S1. Inhibition of GH7 CBHs by cellobiose at 50 ºC.** ^14^C-BC (0.25 mg ml^-1^) was incubated with a mixture of 0.25 µM CBH and 0.025 µM EG (*Tr*Cel5A) at 50 ºC. The concentration of added cellobiose was 0 mM + 0.06 µM *N188*BG (◇), 0 mM (⯁), 0.5 mM (△), 1.0 mM (+), 2.0 mM (○), 5.0 mM (×), 10 mM (□) or 20 mM (*). CBH was (A) *Tr*Cel7A, (B) *Ta*Cel7A, (C) *At*Cel7A, and (D) *Ct*Cel7A.


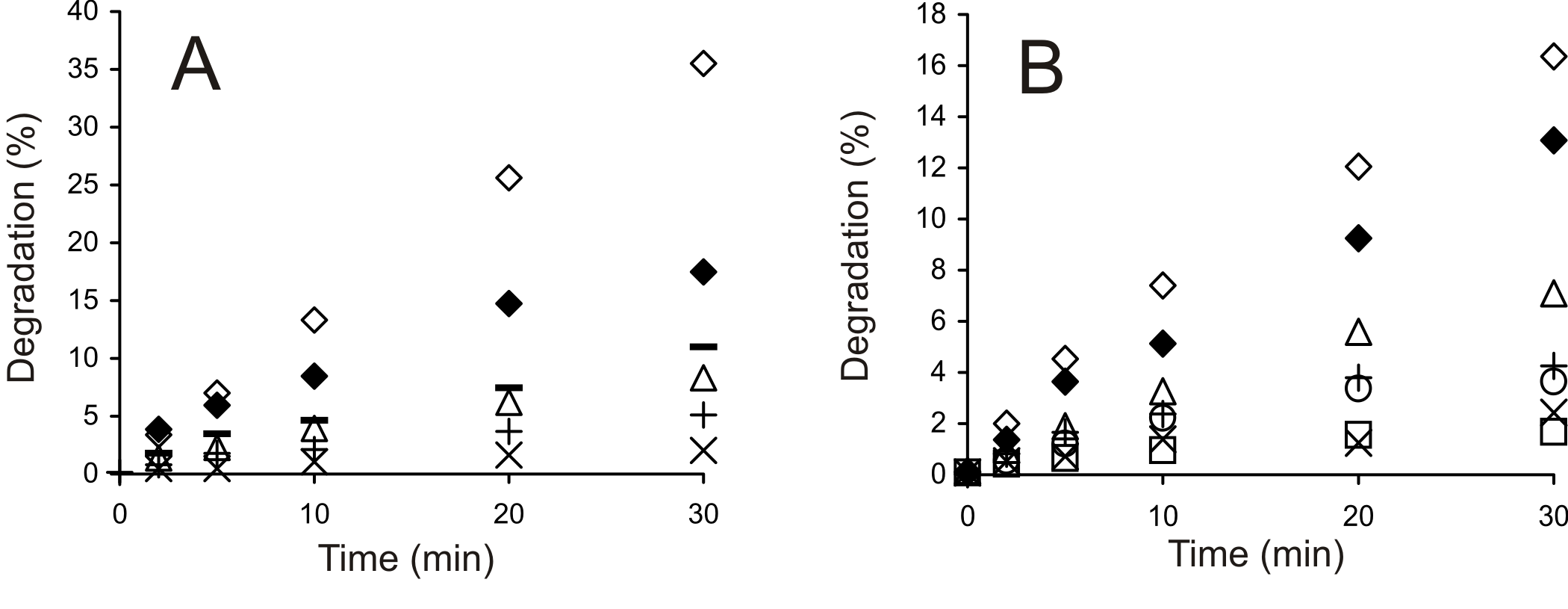


**Figure S2. Inhibition of GH7 CBHs by cellobiose at 25 ºC.** ^14^C-BC (0.25 mg ml^-1^) was incubated with a mixture of 0.25 µM CBH and 0.025 µM EG (*Tr*Cel5A) at 25 ºC. The concentration of added cellobiose was 0 mM + 0.06 µM *N188*BG (◇), 0 mM (⯁), 0.25 mM (-) 0.5 mM (△), 1.0 mM (+), 2.0 mM (○), 5.0 mM (×) or 10 mM (□). CBH was (A) *At*Cel7A, and (B) *Ct*Cel7A.


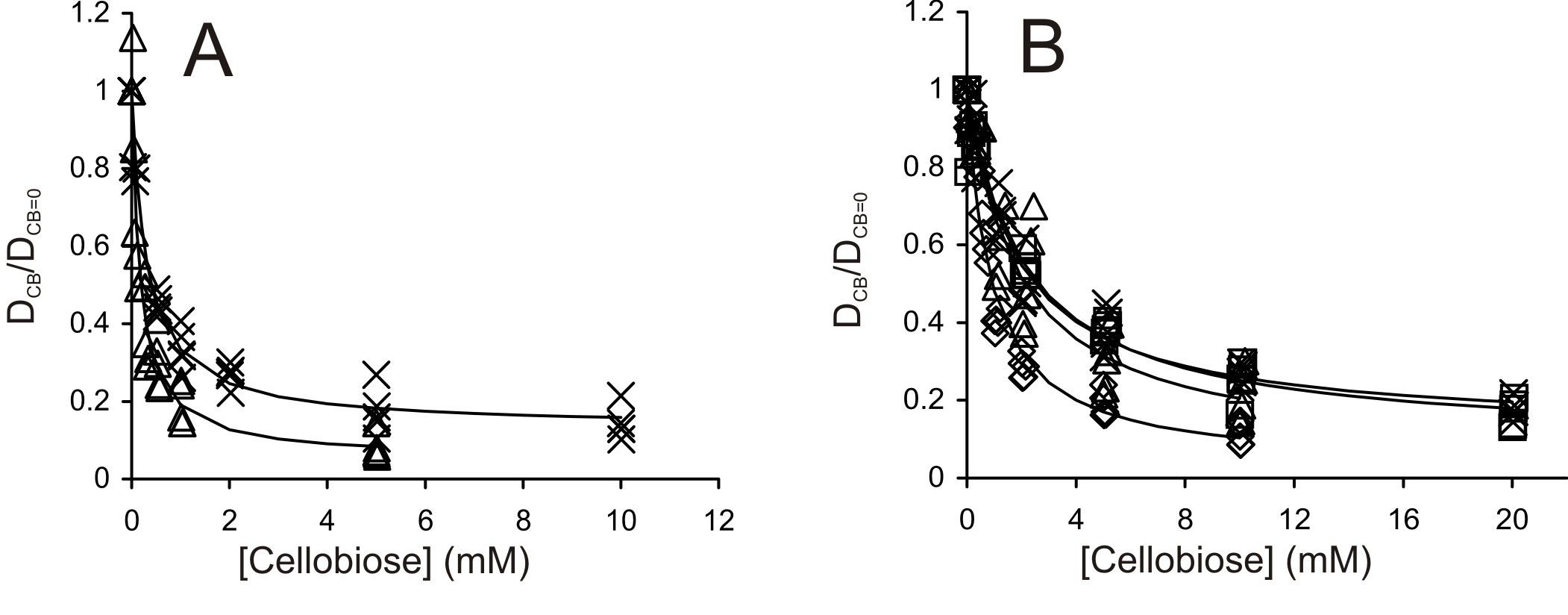


**Figure S3. Analysis of the inhibition of GH7 CBHs by cellobiose at 25 ºC and 50 ºC.** Data for the hydrolysis of ^14^C-BC by the mixture of CBH and *Tr*Cel5A (Figures S1 and S2) were rearranged in the coordinates (D_CB_/D_CB=0_) versus [cellobiose], where D_CB_ and D_CB=0_ represent the degree of conversion of ^14^C-BC in the presence and absence of cellobiose, respectively. (D_CB_/D_CB=0_) values for all hydrolysis time points are shown. CBH was as follows: *Tr*Cel7A (□), *Ta*Cel7A, (◇), *At*Cel7A (△), and *Ct*Cel7A (×). Solid lines are from the non-linear regression according to Equation 5. (A) 25 ºC and (B) 50 ºC.


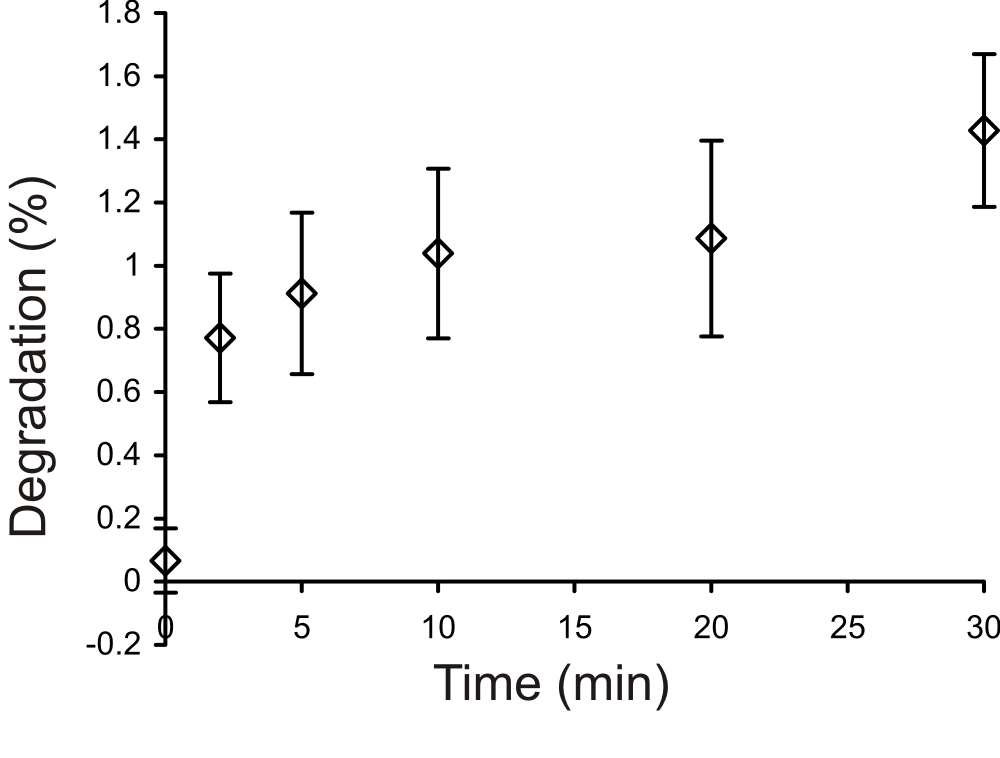


**Figure S4. Hydrolysis of ^14^C-BC by EG, *Tr*Cel5A.** ^14^C-BC (0.25 mg ml^-1^) was incubated with a mixture of 0.025 µM *Tr*Cel5A and 0.06 µM *N188*BG at 25 ºC, 40 ºC, 50 ºC, and 60 ºC. There was no systematic dependency between the activity and temperature within the experiment uncertainty. Therefore, the average values of activity over measurements at different temperatures are plotted.


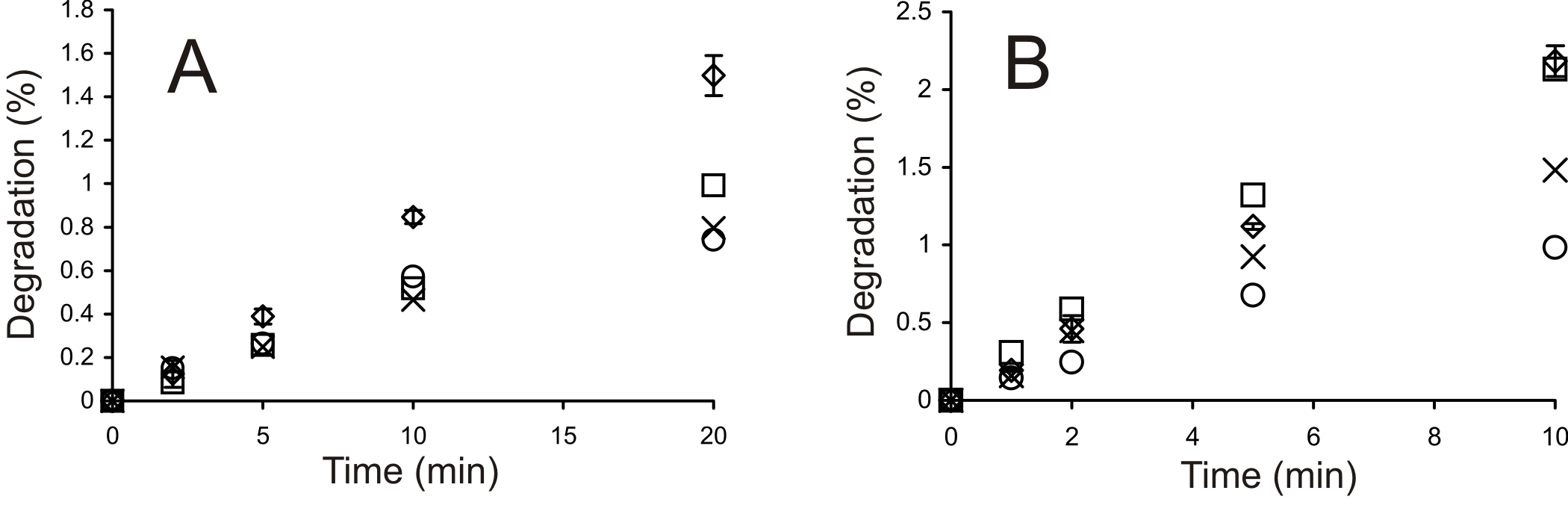


**Figure S5. Inhibition of EGs, *Tr*Cel5A and *Tr*Cel12A, by cellobiose.** ^14^C-amorphous cellulose (0.5 mg ml^-1^) was incubated with 5.0 nM *Tr*Cel5A (panel A) or with 50 nM *Tr*Cel12A (panel B) at 35 ºC. The concentration of added cellobiose was as follows: 0 mM (◇), 75 mM (□), 150 mM (×), or 225 mM (○).
